# Calibrating epigenetic clocks with training data error

**DOI:** 10.1111/eva.13582

**Published:** 2023-07-26

**Authors:** Benjamin Mayne, Oliver Berry, Simon Jarman

**Affiliations:** ^1^ Environomics Future Science Platform, Indian Ocean Marine Research Centre Commonwealth Scientific and Industrial Research Organisation (CSIRO) Crawley Western Australia Australia; ^2^ Curtin University Perth Western Australia Australia

**Keywords:** bioinfomatics/phyloinfomatics, molecular evolution, wildlife management

## Abstract

Animal age data are valuable for management of wildlife populations. Yet, for most species, there is no practical method for determining the age of unknown individuals. However, epigenetic clocks, a molecular‐based method, are capable of age prediction by sampling specific tissue types and measuring DNA methylation levels at specific loci. Developing an epigenetic clock requires a large number of samples from animals of known ages. For most species, there are no individuals whose exact ages are known, making epigenetic clock calibration inaccurate or impossible. For many epigenetic clocks, calibration samples with inaccurate age estimates introduce a degree of error to epigenetic clock calibration. In this study, we investigated how much error in the training data set of an epigenetic clock can be tolerated before it resulted in an unacceptable increase in error for age prediction. Using four publicly available data sets, we artificially increased the training data age error by iterations of 1% and then tested the model against an independent set of known ages. A small effect size increase (Cohen's d >0.2) was detected when the error in age was higher than 22%. The effect size increased linearly with age error. This threshold was independent of sample size. Downstream applications for age data may have a more important role in deciding how much error can be tolerated for age prediction. If highly precise age estimates are required, then it may be futile to embark on the development of an epigenetic clock when there is no accurately aged calibration population to work with. However, for other problems, such as determining the relative age order of pairs of individuals, a lower‐quality calibration data set may be adequate.

## 1 INTRODUCTION

Age is a fundamental parameter for population biology. Animal mortality and fecundity rates are strongly affected by age in all species and collectively can determine population growth rates (Healy et al., 2019). In wildlife management, age information is used to determine the risk of extinction, assist in the management of invasive species and determine the sustainable harvest in fisheries (Bravington, Grewe, & Davies, 2016; Hoenig, 1983; Tabak et al., 2018). Unfortunately, age cannot be determined for most species. Methods such as otolith growth ring analysis or skeletochronology can be used to estimate age in species with growth bands (Campana & Thorrold, 2001; Tomaszewicz et al., 2015). However, these methods are not applicable to all species and can only be carried out on deceased individuals. Bomb radiocarbon has been used to determine age in some long‐lived species and is too expensive to be a viable approach for regular population monitoring (Andrews et al., 2020; Fallon et al., 2019). Ideally, a non‐lethal, affordable and high throughput method would be available to determine age for wildlife management.

Epigenetic ageing has great potential as a wildlife management tool (Jarman et al., 2015). Epigenetic clocks have been developed for a wide range of vertebrate species (Kerepesi et al., 2022; Levine et al., 2020; Mayne, Berry, & Jarman, 2020; Prado et al., 2021; Raj et al., 2021). In some cases, epigenetic age information has already been used for demographic analysis (Riekkola et al., 2018). Epigenetic clocks work by measuring the proportion of cytosine–phosphate–guanine (CpG) nucleotides that have a methyl group at the 5′ position of the cytosine. Age prediction models are calibrated by comparing the DNA methylation levels to known age individuals. DNA methylation can either increase or decrease over the life of an individual at specific CpG sites (Bell et al., 2019; Jylhävä et al., 2017). This occurs in many somatic tissues making epigenetic ageing a potentially non‐lethal method (Horvath, 2013; Stubbs et al., 2017). DNA sequencing has increased in throughput and decreased in cost (Gordon et al., 2020). This gives epigenetic ageing the potential to become a routine method in wildlife management (Frelinger, 2015; Marshall et al., 2017).

Despite the advantages of epigenetic ageing, there are still challenges and limitations to be quantified and addressed. The consequence of errors in ages that are included in training data sets is a potentially important effect that has not been addressed. Under ideal circumstances, epigenetic clocks are calibrated from known ages. A statistical power analysis suggests over 220 samples are ideal for epigenetic clock calibration (Mayne, Berry, & Jarman, 2021). However, for most species, this is a very difficult or impossible sample size to obtain. Numerous phenotypic or chemical measurements can be used as substitutes for an exact age (Mayne, Espinoza, et al., 2021). These alternatives to exact age may have their own levels of error, which may be amplified when in the epigenetic clock. Therefore, it is useful to determine how much error in age data for calibration samples can be tolerated for epigenetic clock calibration. In this study, we systematically increased the age error in four publicly available DNA methylation data sets consisting of known age individuals. We then compared the age error increase model to the baseline model at each iteration to determine the effect of the error increase. Our analysis reveals consistent thresholds where errors in training data overwhelm meaningful predictions from epigenetic clocks.

## 2 METHODS

### 2.1 Known age data sets

This study required a DNA methylation data set with associated accurate ages for each sample. Although it is possible to simulate a DNA methylation data set from database sources, it is not possible to generate a data set with known ages (Rackham et al., 2015). We therefore searched the Gene Expression Omnibus, Short Read Archive, and ArrayExpress for the largest data set with known age sample information (Barrett et al., 2012; Leinonen et al., 2011; Parkinson et al., 2007). Four data sets were used in this study and were chosen as they were from different species (two mammals, one reptile, one fish) and tissues and therefore may capture the variability in developing epigenetic clocks (Table 1). The processed and normalised CpG methylation data for the human and mouse data sets was publicly available and was used in this study, the raw fastq files were only available for the zebrafish and turtle data set. The fastq files were aligned to the zebrafish reference genome (danRer10, Illumina igenomes) and the green sea turtle reference genome (rCheMyd1.pri.v2) with BSSeeker2 v 2.0.3 and bowtie2 v2.3.4 using default settings to obtain DNA methylation values (Guo et al., 2013; Langmead & Salzberg, 2012).

**TABLE 1:** Summary of the four publicly available data sets used in this study.

Reference	Species	Total samples	Tissue type	Age range
(Horvath et al., 2012)	Human	394	Whole blood	16–88 years
(Mayne, Berry, & Jarman, 2020)	Zebrafish	96	Caudal fin	11.9–60.1 weeks
(Reizel et al., 2015)	Mouse	153	Muscle, liver, spleen, and cerebellum	1–31 weeks
(Mayne et al., 2022)	Marine Turtle	63	Flipper Skin	1–43 years

### 2.2 Increasing known age error

The ages in the training data set were increased in error by 1%–100%. This was done by taking the percentage of each age and then randomly adding or subtracting, up to the simulated error level from the actual age. For example, at 10% increased error, the error is increased up to 10%. Therefore, some samples may have their age increased by 5% or decreased by 8% etc., but no more than 10%. For each error increase, a 10‐fold cross‐validation was applied, and the average performance of each model was used for each increase. 100% was chosen to be the maximum as any higher magnitude would result in negative ages. The epigenetic clock models were calibrated using an elastic net regression model (Friedman et al., 2010) a common method for epigenetic clock studies working with a larger number of CpG sites than samples (Horvath, 2013; Mayne et al., 2022; Mayne, Korbie, et al., 2020; Stubbs et al., 2017; Thompson et al., 2017). In each data set, 70% of the samples were randomly split into a training data set and the remaining 30% into a testing data set.

In each iteration, the model was not restricted to the selection of sites used for the complete training model. This was done by setting the α‐parameter to 0.5, thereby shrinking the number of predictors required to the minimum, while maintaining high performance. Although the CpG sites for both human and zebrafish are known, in a new species, they would not be and therefore the analysis here reflect a more real‐world scenario.

### 2.3 Identification of excess error in the training data set

We used absolute and relative error rates as two measurements of epigenetic clock accuracy. We conducted unpaired t‐tests between absolute and relative error rates derived from the baseline model for (chronological age) and derived from models incorporating different levels of error in training age data. Cohen's d was also used to determine the effect between the age error increase and the baseline epigenetic clock model. We used the commonly accepted, small (d = 0.2), medium (d = 0.5) and large (d = 0.8) effect sizes as a guide to determine if the age error increase influenced the performance of age prediction (Cohen, 2013). A linear regression was applied to the mean Cohen's d values between data sets and error increase to generate a model to describe the relationship. All analyses were performed using R v4.2.0 and R scripts are provided in the Data S2.

## 3 RESULTS

### 3.1 Effects of calibration error on epigenetic age error

**FIGURE 1:**
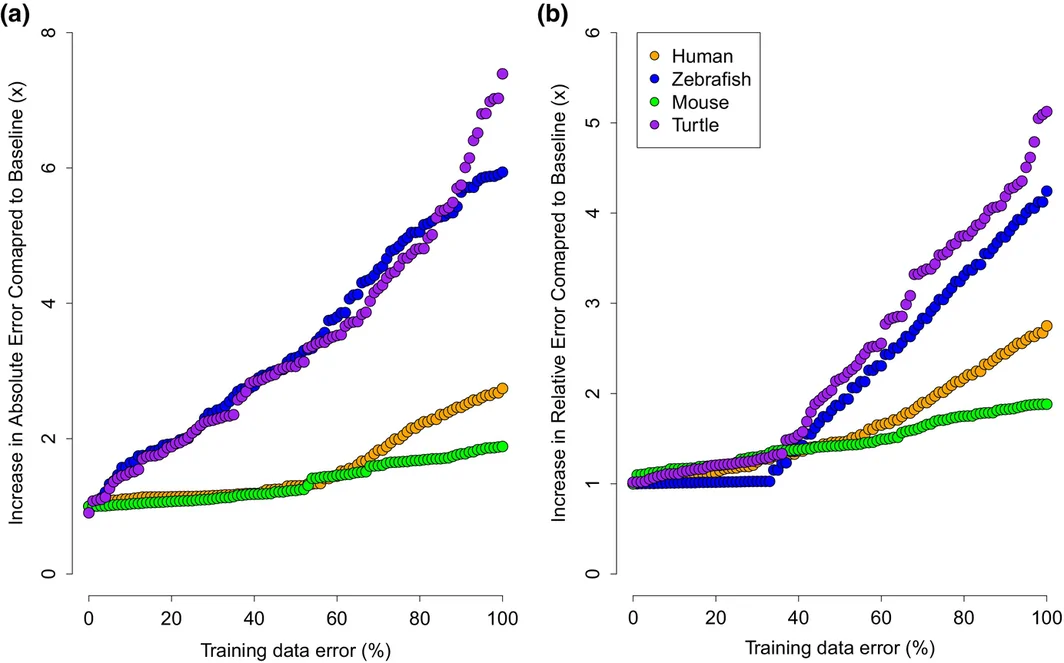
Increase in the (a) absolute and (b) relative error with each iteration increase of error in the training data. The increase in absolute and relative error is compared to the baseline, where no error was introduced in the training data. Orange‐coloured dots represent the human data set, and blue represents the zebrafish data set.

We first determined the baseline performance for each data set by measuring errors in the testing partition. The models produced similar performance to other epigenetic clocks on the same species (Horvath, 2013; Mayne et al., 2022; Mayne, Korbie, et al., 2020; Stubbs et al., 2017). Under our simulated increase in calibration error, the human and mouse data sets increased over two‐fold in absolute and relative error (Table 2, Figure 1a,b), whereas the turtle and zebrafish data sets increased over four‐fold in absolute and relative error (Table 2, Figure 1a,b). The average number of CpG sites selected by the model also decreased with the increased error (Table 2). No overlap between the baseline CpG site selection (no error added to the ages) and at the 100% error increase was found.

**TABLE 2:** Performance of the age prediction models for each species with no additional error in the training data partition (baseline) and with a 100% increase in error.

Species	Baseline				100% increase in training error			
	Correlation	Absolute error	Relative error (%)	CpG sites	Correlation	Absolute error	Relative error (%)	CpG sites
Human	0.97	4.9 years	13	78	0.79	13.5 years	36.2	23
Zebrafish	0.97	3.2 weeks	8.2	30	0.80	18.9 weeks	34.8	15
Mouse	0.97	2.7 weeks	14.3	65	0.79	5.1 weeks	26.9	35
Turtle	0.97	2.7 years	14.1	50	0.79	5.2 years	27	37

*Note*: The correlation in the table refers to the testing partition Pearson correlation.

The smaller sample size data sets of the zebrafish and green turtle exhibited a higher increase in error compared to the human data set (Figure 1). To test whether this was reflective of the total number of samples (394 in human, 96 in zebrafish), the human data set was reduced to a random set of 96 samples, equivalent to the zebrafish data set. We then ran the same process with the 96 samples in training data and found comparable absolute and relative error rates to the zebrafish data set (Figure S1).

### 3.2 Thresholds for the tolerance of error in calibration data

**FIGURE 2:**
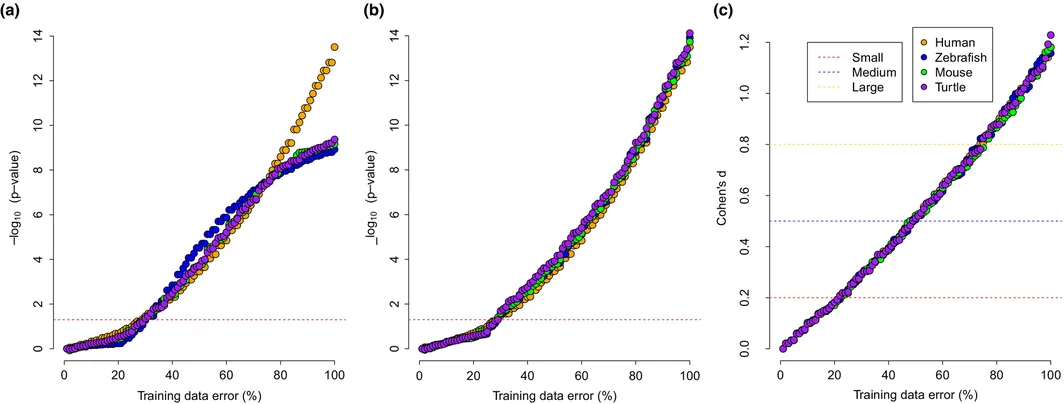
Increase in the error of the training data with three methods of statistical tests. Increase in *p*‐value for *t*‐tests comparing (a) absolute and (b) relative error rate in data with simulated error to the baseline model. The p‐value has been transformed into ‐log_10_ (*p*‐value) to illustrate a positive increase. The horizontal red dotted line represents a *p*‐value = 0.05. (c) Cohen's d increase with training error. The coloured dotted lines show the considered small (0.2), medium (0.5) and large (0.8) effect sizes.

Three methods were used to test the tolerance of error in the calibration data. First, the absolute error increased significantly in all four species when the error was introduced to the training data (Figure 2a). For the human data set, the effect became evident at a 29% additional error (unpaired *t*‐test, *p*‐value = 0.046), 31% for the zebrafish (unpaired *t*‐test, *p*‐value = 0.034), 30% for the mouse (unpaired *t*‐test, *p*‐value = 0.042) and 30% for the turtle (unpaired *t*‐test, *p*‐value = 0.044). Second, the relative error (Figure 2b) significantly increased at 29% additional error for the human data set (unpaired *t*‐test, *p*‐value = 0.047), 31% for the zebrafish (unpaired *t*‐test, *p*‐value = 0.047), 29% for the mouse (unpaired *t*‐test, *p*‐value = 0.039) and 30% for the turtle (unpaired *t*‐test, *p*‐value = 0.020). Third, a small effect size (Cohen's d >0.2) was detectable at 23% for both the human and zebrafish data sets and at 22% for the mouse and turtle data sets (Figure 2c, Table S1). As Cohen's d values were similar between data sets, the mean between data sets was used to generate a linear model with an increase in error. The linear model produced the equation below, where x = training data error %.

$$\text{Effect size}\;\left(\text{Cohe}n^{\prime }s\;d\right)=\frac{1.176x-5.493\;}{100}$$

The equation provides a method to determine the effect size of error increase in the training data. However, it should be noted this was simulated data and the statistical effects we simulated cannot be expected to perfectly reflect real‐world applications.

## 4 DISCUSSION

Epigenetic clocks have the potential to provide important life‐history information for the management of wildlife populations (Steve Horvath et al., 2022). Species that do not have a practical and non‐lethal method to determine age are most likely to benefit as this fundamental life‐history trait has typically been unobtainable. However, one of the most important limitations or uncertainties in epigenetic clock development is the accuracy of age estimates for samples used to train the clock model. Although alternatives to exact ages may be used, the degree of error associated with the estimated ages will limit the accuracy of the clock being developed. The error will be amplified in the epigenetic clock, potentially generating a poor predictor of true age. Despite the sample size differences in the data sets, a similar absolute error rate was observed with increasing calibration data error. It also suggests that sample size has a contributing role in the overall error rate in the epigenetic model. The human data set has a sufficient sample size (>220), whereas the zebrafish and turtle data are reflective of a lower sample size data set (Mayne, Berry, & Jarman, 2021). This suggests the sample size of the data set is independent of the increase in error. Our simulation of increasing the training data set error in a stepwise manner found 22% is the maximum error that can be tolerated before a statistically significant difference is detected from models developed with exact age. Although sample size can impact the performance of an epigenetic clock, the error in the training data impacts model predictions irrespective of sample size (Mayne, Berry, & Jarman, 2021).

A limitation of this study is the use of only four data sets. Although many DNA methylation datasets are publicly available, most do not have the accompanying phenotype data, and critically, age information. This makes it difficult to conduct meta‐analyses and to do simulation studies. This also limits our ability to test other factors that may contribute to error or need to be accounted for in epigenetic clock models. For example, biological factors such as sex and population of origin can exhibit differential DNA methylation patterns (Bain et al., 2021; Hu & Barrett, 2017). Biological factors other than age are rarely accounted for in epigenetic clocks, except for sex in mammal studies (Horvath, 2013; Mayne et al., 2017; Stubbs et al., 2017). This likely reflects the difficulty of sourcing sufficient samples with accurately determined ages to adequately account for other factors. However, our use of four different species from three vertebrate classes and with multiple tissue types still showed a similar trend with the effect size. Therefore, the error introduced into the training data may act independently of other biological factors.

Our focus has been on identifying attributes of calibration data sets that reduce the capacity of epigenetic clocks to accurately predict age. We applied error across entire data sets, but in real‐life data sets, there may be more idiosyncratic patterns of error, including individuals with very large errors. These errors may be difficult to identify or unknown. One possible method to identify error is to generate the model and if a few selected samples have high error rates, they should be investigated further. If any outliers have been removed the model can be regenerated, this process can be repeated until a final model is produced.

It must be recognised that high‐accuracy age estimates are not a requirement for all downstream wildlife management applications. Indeed, in some cases, relative ages will suffice. For example, life tables are generally presented with annualised age estimates or equivalent intervals for shorter‐lived organisms (Hsieh, 1991). In this case, it is beneficial to have the accuracy of age prediction calibrated to these intervals otherwise inaccurate estimates of survivorship, mortality rates and life expectancies will result (Deevey, 1947; Mayne, Berry, & Jarman, 2020). On the other hand, analyses based on pedigrees, such as close kin capture‐recapture, only require the age order of pairs of individuals to accurately distinguish alternative kinship relationships (Bravington, Skaug, & Anderson, 2016). In this application, the tolerance of error in age estimation models may be greater and correspond to increased tolerance for error in calibration data.

Another perspective on the value of epigenetic clocks derived from error‐prone training data is that for most wild organisms, age data are difficult to obtain and researchers may not have the freedom to be highly selective in curating calibration data. Our analyses provide useful rules of thumb for researchers considering the development of epigenetic clocks and show that when calibration data is likely to exceed 22% error in exact ages of individuals, the performance of the epigenetic clock will be sub‐optimal. For some applications that require high accuracy, this may dictate that no further development is warranted. In other, less stringent cases, however, researchers might proceed with the understanding that the accuracy of the epigenetic clock will be sub‐optimal, with our simulation results providing approximations of the extent of inaccuracy introduced. In such cases, researchers should also be prompted to consider periodic recalibration of epigenetic clocks as training samples with more accurate age become available.

## CONFLICT OF INTEREST STATEMENT

The authors have no conflicts of interest to declare.

## Supporting information


Data S1:

Data S2:

## Data Availability

The data used in this study was publicly available data. The human (GSE41037) and mouse (GSE60012) studies can be found on NCBI Gene Expression Omnibus by searching the respective GSE accession numbers. The zebrafish and turtle data can be found in the CSIRO's data repository https://doi.org/10.25919/5f63ce026960a and https://doi.org/10.25919/mp9e‐6277). Data sharing is not applicable to this article as no new data were created or analysed in this study.
