# EXPRESSION OF CONCERN: Calibrating Epigenetic Clocks with Training Data Error

**DOI:** 10.1111/eva.70343

**Published:** 2026-10-30

## Abstract

**EXPRESSION OF CONCERN**: MayneB. , BerryO. and JarmanS. , “Calibrating Epigenetic Clocks with Training Data Error,” Evolutionary Applications 16, no. 8 (2023): 1496–1502, 10.1111/eva.13582.PMC1044508637622096

This Expression of Concern is for the above article, published online on 26 July 2023 in Wiley Online Library (wileyonlinelibrary.com), and has been issued by agreement between the journal Editor‐in‐Chief, Maren Wellenreuther; and John Wiley & Sons Ltd. The Expression of Concern has been agreed since there are currently difficulties in replicating the relevant data. Therefore, the journal has decided to issue an Expression of Concern to alert readers to these concerns while this matter remains under investigation.

