# Supplementary material for: Calibrating epigenetic clocks with training data error
Source: Evol Appl. 2023 Jul 26;16(8):1496–502. doi: 10.1111/eva.13582 (PMC10445086; doi:10.1111/eva.13582)
Supplement: Supplementary file 1 — Data S1: [file EVA-16-1496-s002.docx]

**Supplemental Information for:**

**Calibrating epigenetic clocks with training data error**

**Table of Contents:**

| **Supplementary Results** | Page 2 |
| --- | --- |
| Supplementary Table 1 | Page 2 |
| Supplementary Figure 1 | Page 5 |

**Supplementary Results**

**Table 1.** Increase in training data error and the performance in the testing dataset for all four species. Correlation was determined

|  | **Human** | | **Zebrafish** | | **Mouse** | | **Turtle** | |
| --- | --- | --- | --- | --- | --- | --- | --- | --- |
| **Error (%)** | **Correlation** | **Cohen's d** | **Correlation** | **Cohen's d** | **Correlation** | **Cohen's d** | **Correlation** | **Cohen's d** |
| Baseline | 0.968002 | NA | 0.966681 | NA | 0.96939 | NA | 0.96939 | NA |
| 1 | 0.966711 | 0 | 0.963054 | 0 | 0.967986 | 0 | 0.967986 | 0 |
| 2 | 0.965816 | 0.021132 | 0.961672 | 0.021234 | 0.964922 | 0.020458 | 0.964922 | 0.020448 |
| 3 | 0.965752 | 0.021132 | 0.961604 | 0.022042 | 0.96479 | 0.021171 | 0.96479 | 0.02217 |
| 4 | 0.965444 | 0.033343 | 0.960634 | 0.032909 | 0.964785 | 0.032106 | 0.964785 | 0.031904 |
| 5 | 0.964873 | 0.033343 | 0.959732 | 0.033588 | 0.963197 | 0.033552 | 0.963197 | 0.034388 |
| 6 | 0.963276 | 0.060407 | 0.958244 | 0.058245 | 0.961732 | 0.058935 | 0.961732 | 0.058239 |
| 7 | 0.959928 | 0.060407 | 0.957924 | 0.062574 | 0.960827 | 0.061723 | 0.960827 | 0.059805 |
| 8 | 0.959891 | 0.073392 | 0.957266 | 0.073361 | 0.958897 | 0.070458 | 0.958897 | 0.071141 |
| 9 | 0.959825 | 0.073392 | 0.956314 | 0.075335 | 0.95723 | 0.076779 | 0.95723 | 0.075897 |
| 10 | 0.95735 | 0.099658 | 0.953867 | 0.099187 | 0.955054 | 0.102281 | 0.955054 | 0.097338 |
| 11 | 0.949657 | 0.099658 | 0.948457 | 0.102766 | 0.954468 | 0.104123 | 0.954468 | 0.10185 |
| 12 | 0.946395 | 0.112186 | 0.94372 | 0.10947 | 0.953886 | 0.110035 | 0.953886 | 0.107509 |
| 13 | 0.946212 | 0.112186 | 0.937354 | 0.115602 | 0.953097 | 0.115761 | 0.953097 | 0.11213 |
| 14 | 0.944633 | 0.136073 | 0.931757 | 0.134089 | 0.95281 | 0.138108 | 0.95281 | 0.134085 |
| 15 | 0.944175 | 0.136073 | 0.928733 | 0.141925 | 0.952344 | 0.142242 | 0.952344 | 0.142457 |
| 16 | 0.943639 | 0.147509 | 0.92738 | 0.146157 | 0.952211 | 0.145149 | 0.952211 | 0.149349 |
| 17 | 0.941997 | 0.147509 | 0.927155 | 0.146901 | 0.951176 | 0.154202 | 0.951176 | 0.149937 |
| 18 | 0.94064 | 0.160616 | 0.925847 | 0.15521 | 0.950658 | 0.156801 | 0.950658 | 0.157604 |
| 19 | 0.940021 | 0.173316 | 0.919228 | 0.172606 | 0.95049 | 0.167517 | 0.95049 | 0.171681 |
| 20 | 0.936135 | 0.173316 | 0.916443 | 0.178988 | 0.948039 | 0.170233 | 0.948039 | 0.181913 |
| 21 | 0.935872 | 0.185967 | 0.914133 | 0.188734 | 0.943645 | 0.186155 | 0.943645 | 0.19257 |
| 22 | 0.935835 | 0.199398 | 0.914094 | 0.192994 | 0.941514 | 0.207852 | 0.941514 | 0.208441 |
| 23 | 0.933075 | 0.213089 | 0.91266 | 0.207976 | 0.939545 | 0.207925 | 0.939545 | 0.219102 |
| 24 | 0.931458 | 0.213089 | 0.911268 | 0.220419 | 0.938477 | 0.213323 | 0.938477 | 0.219744 |
| 25 | 0.928605 | 0.227944 | 0.906924 | 0.23197 | 0.937338 | 0.217606 | 0.937338 | 0.222653 |
| 26 | 0.926892 | 0.24533 | 0.903656 | 0.246596 | 0.936443 | 0.243315 | 0.936443 | 0.251761 |
| 27 | 0.925983 | 0.261518 | 0.901674 | 0.250923 | 0.935634 | 0.257523 | 0.935634 | 0.25612 |
| 28 | 0.921974 | 0.261518 | 0.898646 | 0.262286 | 0.934975 | 0.266755 | 0.934975 | 0.274175 |
| 29 | 0.917833 | 0.276963 | 0.894104 | 0.287453 | 0.927779 | 0.270776 | 0.927779 | 0.274236 |
| 30 | 0.916427 | 0.292866 | 0.891301 | 0.292425 | 0.927106 | 0.28919 | 0.927106 | 0.294526 |
| 31 | 0.913013 | 0.307529 | 0.889882 | 0.301904 | 0.925842 | 0.304149 | 0.925842 | 0.302007 |
| 32 | 0.91096 | 0.307529 | 0.884916 | 0.304001 | 0.924267 | 0.311571 | 0.924267 | 0.303338 |
| 33 | 0.910101 | 0.307529 | 0.884318 | 0.319402 | 0.9238 | 0.313361 | 0.9238 | 0.305764 |
| 34 | 0.909142 | 0.338112 | 0.883344 | 0.32724 | 0.920393 | 0.339968 | 0.920393 | 0.326505 |
| 35 | 0.902895 | 0.338112 | 0.883023 | 0.339836 | 0.920207 | 0.344516 | 0.920207 | 0.340892 |
| 36 | 0.902522 | 0.351937 | 0.881973 | 0.354142 | 0.918612 | 0.34946 | 0.918612 | 0.368484 |
| 37 | 0.900512 | 0.351937 | 0.881823 | 0.363656 | 0.91765 | 0.350273 | 0.91765 | 0.368981 |
| 38 | 0.893199 | 0.381319 | 0.881249 | 0.366895 | 0.916232 | 0.373938 | 0.916232 | 0.369165 |
| 39 | 0.893083 | 0.381319 | 0.879733 | 0.379028 | 0.914182 | 0.3808 | 0.914182 | 0.382925 |
| 40 | 0.89087 | 0.394914 | 0.878667 | 0.391874 | 0.907814 | 0.384306 | 0.907814 | 0.390345 |
| 41 | 0.889481 | 0.394914 | 0.877298 | 0.396909 | 0.906849 | 0.41112 | 0.906849 | 0.401871 |
| 42 | 0.888997 | 0.424811 | 0.875917 | 0.427301 | 0.903645 | 0.412531 | 0.903645 | 0.41501 |
| 43 | 0.882571 | 0.424811 | 0.874722 | 0.429284 | 0.903141 | 0.421202 | 0.903141 | 0.423504 |
| 44 | 0.880355 | 0.440249 | 0.872817 | 0.43507 | 0.90197 | 0.437112 | 0.90197 | 0.4258 |
| 45 | 0.877979 | 0.456997 | 0.87149 | 0.451421 | 0.895444 | 0.440211 | 0.895444 | 0.451019 |
| 46 | 0.874243 | 0.472113 | 0.867332 | 0.454628 | 0.895118 | 0.470229 | 0.895118 | 0.466544 |
| 47 | 0.873485 | 0.472113 | 0.867175 | 0.461236 | 0.892965 | 0.493025 | 0.892965 | 0.471459 |
| 48 | 0.87307 | 0.487823 | 0.865214 | 0.493874 | 0.89208 | 0.500603 | 0.89208 | 0.482754 |
| 49 | 0.870066 | 0.505507 | 0.864709 | 0.497665 | 0.891807 | 0.504025 | 0.891807 | 0.501379 |
| 50 | 0.869206 | 0.505507 | 0.860214 | 0.506496 | 0.891666 | 0.52161 | 0.891666 | 0.511052 |
| 51 | 0.868748 | 0.522394 | 0.858709 | 0.522868 | 0.890977 | 0.527391 | 0.890977 | 0.519891 |
| 52 | 0.86685 | 0.522394 | 0.857408 | 0.523724 | 0.887086 | 0.527805 | 0.887086 | 0.532398 |
| 53 | 0.862951 | 0.555609 | 0.857151 | 0.546064 | 0.88536 | 0.533874 | 0.88536 | 0.547522 |
| 54 | 0.862476 | 0.555609 | 0.857116 | 0.555361 | 0.882674 | 0.541592 | 0.882674 | 0.569593 |
| 55 | 0.862198 | 0.571197 | 0.852502 | 0.567023 | 0.882466 | 0.563935 | 0.882466 | 0.574937 |
| 56 | 0.861463 | 0.571197 | 0.851196 | 0.578544 | 0.881129 | 0.571112 | 0.881129 | 0.57996 |
| 57 | 0.859866 | 0.603553 | 0.848863 | 0.578725 | 0.87819 | 0.605001 | 0.87819 | 0.593271 |
| 58 | 0.859759 | 0.603553 | 0.84799 | 0.592221 | 0.876827 | 0.606725 | 0.876827 | 0.593491 |
| 59 | 0.8584 | 0.620502 | 0.847869 | 0.604769 | 0.87676 | 0.609156 | 0.87676 | 0.606656 |
| 60 | 0.854271 | 0.620502 | 0.847086 | 0.615888 | 0.872805 | 0.629789 | 0.872805 | 0.641439 |
| 61 | 0.853116 | 0.655265 | 0.845493 | 0.644945 | 0.871918 | 0.642795 | 0.871918 | 0.643245 |
| 62 | 0.852836 | 0.655265 | 0.84516 | 0.649911 | 0.870007 | 0.649554 | 0.870007 | 0.660968 |
| 63 | 0.852509 | 0.672067 | 0.843758 | 0.669846 | 0.869439 | 0.668532 | 0.869439 | 0.67733 |
| 64 | 0.851212 | 0.672067 | 0.840034 | 0.672516 | 0.865816 | 0.674107 | 0.865816 | 0.685695 |
| 65 | 0.84827 | 0.688602 | 0.838411 | 0.677266 | 0.864809 | 0.681679 | 0.864809 | 0.700527 |
| 66 | 0.845869 | 0.704973 | 0.837245 | 0.679633 | 0.86381 | 0.689546 | 0.86381 | 0.702293 |
| 67 | 0.843462 | 0.704973 | 0.836372 | 0.687373 | 0.85995 | 0.706141 | 0.85995 | 0.702432 |
| 68 | 0.841851 | 0.721116 | 0.836291 | 0.712432 | 0.859915 | 0.707974 | 0.859915 | 0.7256 |
| 69 | 0.841277 | 0.73678 | 0.836146 | 0.740579 | 0.853646 | 0.713461 | 0.853646 | 0.732772 |
| 70 | 0.839626 | 0.75374 | 0.834591 | 0.742111 | 0.852225 | 0.728815 | 0.852225 | 0.733424 |
| 71 | 0.839572 | 0.75374 | 0.830749 | 0.777658 | 0.851725 | 0.744824 | 0.851725 | 0.765135 |
| 72 | 0.836854 | 0.771289 | 0.829835 | 0.781892 | 0.847716 | 0.755759 | 0.847716 | 0.76835 |
| 73 | 0.833001 | 0.788724 | 0.827967 | 0.784525 | 0.842873 | 0.764448 | 0.842873 | 0.775594 |
| 74 | 0.830649 | 0.806881 | 0.824598 | 0.822844 | 0.84148 | 0.78116 | 0.84148 | 0.780834 |
| 75 | 0.829362 | 0.806881 | 0.822622 | 0.827441 | 0.839927 | 0.787288 | 0.839927 | 0.824288 |
| 76 | 0.826168 | 0.824839 | 0.82261 | 0.832262 | 0.839021 | 0.822056 | 0.839021 | 0.836317 |
| 77 | 0.825996 | 0.843179 | 0.820524 | 0.835942 | 0.837139 | 0.822968 | 0.837139 | 0.837253 |
| 78 | 0.82542 | 0.862798 | 0.820476 | 0.837822 | 0.836877 | 0.860977 | 0.836877 | 0.868343 |
| 79 | 0.823279 | 0.862798 | 0.819782 | 0.867565 | 0.835617 | 0.865154 | 0.835617 | 0.876272 |
| 80 | 0.823241 | 0.882729 | 0.819705 | 0.876103 | 0.833591 | 0.866007 | 0.833591 | 0.877587 |
| 81 | 0.823007 | 0.901739 | 0.81787 | 0.881995 | 0.833011 | 0.888425 | 0.833011 | 0.878367 |
| 82 | 0.822548 | 0.901739 | 0.815669 | 0.885702 | 0.830565 | 0.890293 | 0.830565 | 0.922626 |
| 83 | 0.820249 | 0.921734 | 0.814953 | 0.938475 | 0.827048 | 0.905281 | 0.827048 | 0.924972 |
| 84 | 0.819636 | 0.921734 | 0.813884 | 0.940445 | 0.824975 | 0.9163 | 0.824975 | 0.94505 |
| 85 | 0.819376 | 0.959718 | 0.813801 | 0.952992 | 0.822562 | 0.924422 | 0.822562 | 0.949095 |
| 86 | 0.81709 | 0.959718 | 0.81366 | 0.98143 | 0.821805 | 0.926592 | 0.821805 | 0.952306 |
| 87 | 0.817085 | 0.979598 | 0.813655 | 0.998449 | 0.821282 | 0.952297 | 0.821282 | 0.962835 |
| 88 | 0.816864 | 0.998416 | 0.813248 | 0.999438 | 0.813977 | 0.971098 | 0.813977 | 1.003522 |
| 89 | 0.813827 | 1.01896 | 0.811403 | 1.01248 | 0.812824 | 0.981403 | 0.812824 | 1.004183 |
| 90 | 0.813249 | 1.01896 | 0.809625 | 1.015789 | 0.80801 | 1.021417 | 0.80801 | 1.019677 |
| 91 | 0.810756 | 1.039397 | 0.809585 | 1.016095 | 0.805172 | 1.035764 | 0.805172 | 1.04189 |
| 92 | 0.810715 | 1.057938 | 0.808025 | 1.026641 | 0.805139 | 1.058461 | 0.805139 | 1.058704 |
| 93 | 0.806745 | 1.079014 | 0.80785 | 1.084658 | 0.803166 | 1.058673 | 0.803166 | 1.062289 |
| 94 | 0.803496 | 1.079014 | 0.807074 | 1.088483 | 0.802822 | 1.068029 | 0.802822 | 1.080272 |
| 95 | 0.801868 | 1.100397 | 0.801602 | 1.113649 | 0.802446 | 1.069365 | 0.802446 | 1.084064 |
| 96 | 0.797592 | 1.119702 | 0.801199 | 1.127755 | 0.800552 | 1.094434 | 0.800552 | 1.096259 |
| 97 | 0.794509 | 1.119702 | 0.799079 | 1.140492 | 0.798226 | 1.098592 | 0.798226 | 1.104724 |
| 98 | 0.792645 | 1.140646 | 0.798633 | 1.150221 | 0.796505 | 1.160461 | 0.796505 | 1.137492 |
| 99 | 0.791875 | 1.140646 | 0.798363 | 1.152891 | 0.79161 | 1.167766 | 0.79161 | 1.192475 |
| 100 | 0.79128 | 1.181433 | 0.795002 | 1.157511 | 0.791187 | 1.180558 | 0.791187 | 1.228436 |


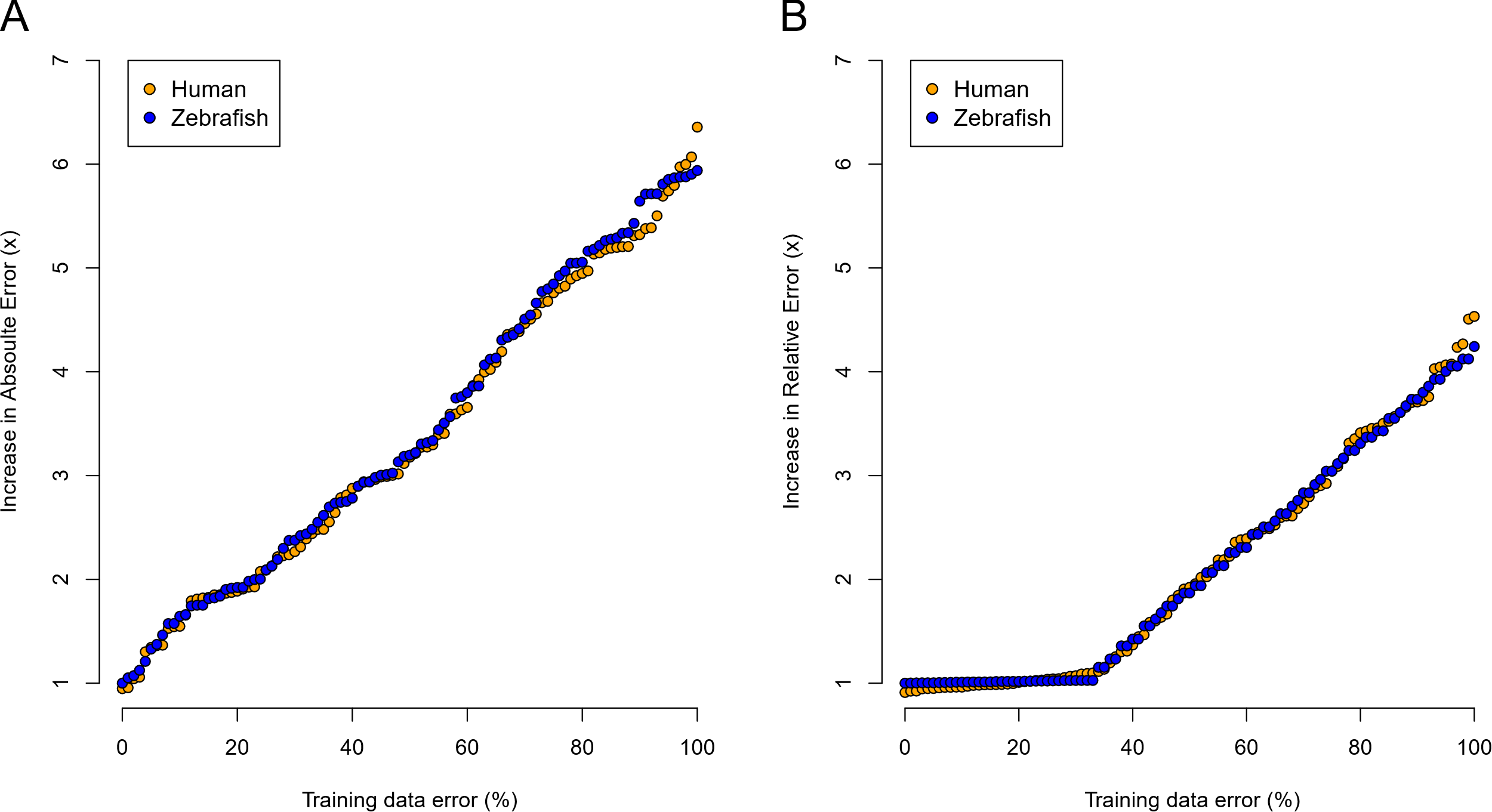


**Supplementary Figure 2.** Random selection of 96 human samples in comparison to the zebrafish samples. The **A.** absolute and **B.** relative error rates between both datasets are similar with increasing calibration dataset error when the sample sizes are the same.
